# Epidemiological Analysis of Extended-Spectrum β-Lactamase-Producing *Klebsiella pneumoniae* Outbreak in a Neonatal Clinic in Poland

**DOI:** 10.3390/antibiotics12010050

**Published:** 2022-12-28

**Authors:** Agata Pruss, Paweł Kwiatkowski, Helena Masiuk, Iwona Bilska, Stefania Giedrys-Kalemba, Barbara Dołęgowska

**Affiliations:** 1 Department of Laboratory Medicine, Pomeranian Medical University in Szczecin, Powstancow Wielkopolskich 72, 70-111 Szczecin, Poland; 2 Department of Diagnostic Immunology, Pomeranian Medical University in Szczecin, Powstancow Wielkopolskich 72, 70-111 Szczecin, Poland; 3 Department of Medical Microbiology, Pomeranian Medical University in Szczecin, Powstancow Wielkopolskich 72, 70-111 Szczecin, Poland; 4 Microbiological Laboratory, Independent Public Clinical Hospital No. 1 in Szczecin, Unii Lubelskiej 1, 71-252 Szczecin, Poland

**Keywords:** neonates, outbreak, neonatal clinic, extended-spectrum β-lactamase-producing *Klebsiella pneumoniae*

## Abstract

*Klebsiella pneumoniae* is one of the most common etiological agents isolated from epidemic outbreaks in neonatal wards. We describe how an extended-spectrum β-lactamase-producing *K. pneumoniae* (ESBL-KP) outbreak in a neonatal ward was extinguished. During the outbreak, which lasted over two months, 26 neonates were tested for *K. pneumoniae*, and 42 environmental swabs were taken. Drug susceptibility was determined for the isolated strains, and their virulence and phylogenetic similarity were checked. ESBL-KP colonization was confirmed in 18 neonates, and six were also confirmed to be infected. All strains isolated from patients represented one clonal type, *K. pneumoniae.* One strain isolated from an environmental source was determined to be a unique pulsed-field gel electrophoresis pattern. Gestational age and Apgar score were assessed as statistically significant for neonates with ESBL-KP infection. The epidemiological measures taken have been successful, and no further cases appeared. Immediate tightening of hospital hygiene rules, screening of all hospitalized neonates, and cohorting ESBL-KP-positive patients proved effective in controlling and ending the outbreak. The lack of ESBL-KP in the environment suggests that the outbreak was transmitted by colonized hospital staff. This theory could be confirmed by introducing mandatory screening for medical personnel.

## 1. Introduction

*Klebsiella pneumoniae*, a representative strain of gut microbiota, is a Gram-negative rod causing a wide range of extraintestinal infections. A great variety of factors determine its virulence. Pneumonia, bloodstream, or urinary tract infections (UTIs) caused by *K. pneumoniae* are the most frequently reported infections in neonatal intensive care units (NICUs). Especially dangerous are infections caused by multidrug-resistant *K. pneumoniae* producing extended-spectrum β-lactamase (ESBL) or carbapenem hydrolyzing β-lactamase.

Asymptomatic carriers of *K. pneumoniae* act as reservoirs contributing to the nosocomial spread of these bacteria from patient to patient [1]. Multidrug resistance allows such organisms to persist both in the gastrointestinal tract of staff or patients in the hospital environment and significantly reduces the therapeutic options; thus, *K. pneumoniae* poses a severe threat to public health [2]. Implementation of adequate infection and control strategies such as rational antibiotic therapy, colonization screening, disinfection, and isolation policies aims to minimize the spread of infection and successfully control the outbreak. Determining clonal relatedness of isolated bacteria aims to track the path of transmission, identify the source of an etiological agent, and therefore predict and prevent further emergence of a pathogen from the source [3].

The study aimed to describe the retrospective analysis of the ESBL-producing *K. pneumoniae* (ESBL-KP) outbreak in the Polish Neonatal Clinic (NC), rapidly interrupting hospital hygiene intervention.

## 2. Results

### 2.1. Outbreak Description

**Figure 1:**
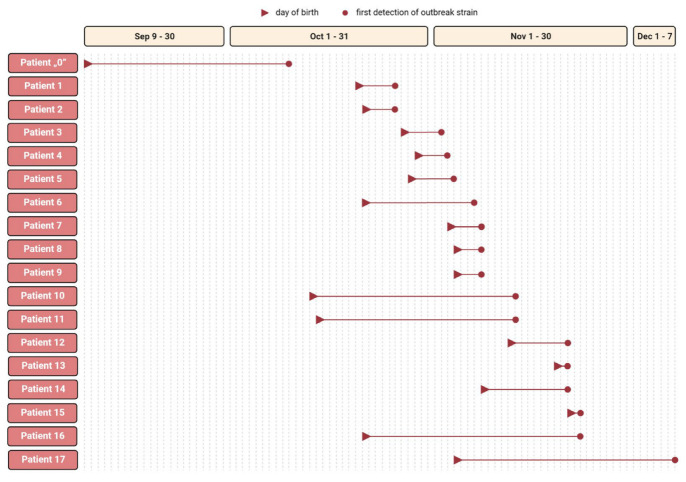
Outbreak timeline.

An ESBL-KP outbreak lasted in NC for over two months. In this period, 26 neonates were hospitalized. In 18 (69.2%) neonates, oral or rectal colonization or both with ESBL-KP was confirmed. Among the neonates, 12 demonstrated symptoms of ongoing infection. ESBL-KP infection was diagnosed in six neonates (positive clinical specimen and rectal/oral colonization together with symptoms of infection). Sepsis was recognized in two cases, and urinary tract infection was found in another two cases. Concurrently, in one case, urosepsis and meningitis were present, and in another case, conjunctivitis and pneumonia were found. Probable infection (pneumonia) was determined in six neonates who demonstrated raised temperature and tachypnea and were rectal/oral ESBL-KP-positive. These infections were not confirmed microbiologically. All infected neonates received meropenem with gentamicin; four required mechanical ventilation. No fatal onset during the outbreak period was reported. All patients were successfully treated and discharged. Characteristics of the patients are in Table 1, and the outbreak timeline is in Figure 1.

### 2.2. Chronological Case Analysis

The index case (patient „0”) was an 11-day-old male neonate that was transferred from the obstetrics/gynecology ward of a provincial hospital to NC due to respiratory disorders and the symptoms of pneumonia. He was born via Cesarean section at 38 weeks of gestation after the mother developed preeclampsia, with a birth weight of 2930 g and Apgar scores of 10, 10, and 10. The patient did not require mechanical ventilation. Empirically, meropenem with gentamicin was administrated. On the first day of hospitalization screening swabs were taken, and the presence of ESBL-KP was confirmed. No specimen for microbiological examination confirming the etiology of pneumonia was collected. After 14 days of hospitalization the patient was discharged. It is supposed that patient „0” was the most probable reservoir of the ESBL-KP outbreak in NC (hypothesis).

After two weeks (26 October) in patient 1, symptoms of conjunctivitis and pneumonia were observed. Conjunctival and rectal swabs cultures revealed the presence of ESBL-KP.

Patient 2 was admitted to NC from another hospital with pneumonia. After two days, rectal colonization with ESBL-KP was confirmed. On the 6 day of hospitalization, the neonate developed symptoms of urosepsis. In both blood and urine samples, the presence of ESBL-KP was detected. On the 29 day of hospitalization, meningitis was confirmed.

On 2 November, simultaneous rectal colonization and blood-positive culture infection were confirmed in patient 3, whereas patient 4 demonstrated symptoms of UTI. The next day a positive urine culture and rectal and oral cavity colonization were confirmed. In patient 5, rectal and oral cavity colonization was confirmed on 4 November, but no symptoms of infection were observed until the day of discharge.

On 7 November, rectal colonization was confirmed in patient 6, who developed symptoms of blood ESBL-KP infection and pneumonia the next day.

On 8 November, rectal/oral cavity colonization was confirmed in patient 7, patient 8, and patient 9. In the following days, pneumonia was recognized in patient 7 and patient 8.

On 13 November, rectal colonization was confirmed in patient 10 and patient 11, who developed symptoms of pneumonia.

On 21 November, rectal colonization was confirmed in patient 12 and patient 13 without symptoms of infection until the day of discharge. Patient 14, with oral cavity and rectal colonization, developed pneumonia.

On 7 December, rectum colonization and positive urine culture were confirmed in patient 17. Detailed data are presented in Table 1. There were the last isolates of ESBL-KP in neonates during the described outbreak in NC.

The subsequent single isolates of ESBL-KP from neonates in this NC were confirmed after 6, 8, and 10 months. All belonged to other genetic clones.

### 2.3. Data Analyzed

A set of data of infected or colonized neonates or both was analyzed. Data included: gender, birth weight, gestational course and age, and type of delivery. Gestational age and Apgar score were assessed as statistically significant (*p* < 0.05) with ESBL-KP infection, whereas other clinical variables were not identified as risk factors (Table 2).

### 2.4. Environmental Contamination Control

All environmental samples collected during the outbreak were negative for ESBL-KP except one of four swabs taken from the bathtub. On stethoscopes, tape measure, three changing tables, and buttons, the presence of coagulase-negative staphylococci (CNS), *Micrococcus* spp., and aerobic spore-forming rods were isolated. A point source remained unidentified by investigating the environmental samples. Microbiological cultures of swabs collected from medical sanitation (all sinks in delivery rooms, adjacent rooms, kitchen, and ambulatory rooms) were sterile or contaminated with microorganisms with the lack of relevance concerning *K. pneumoniae.*

### 2.5. Microbiology and Molecular Results

All isolates showed the same phenotypic screening confirmed ESBL production and were resistant to penicillin with inhibitors, cephalosporins, and trimethoprim-sulfamethoxazole. Strains were susceptible only to gentamicin, amikacin, and meropenem. PCR showed that all isolates carried *bla*_TEM_ and *bla*_CTX-M_ genes. All strains also showed the presence of *fimH*, *wabg*, *uge*, *kfu* and *iroN*.

### 2.6. Pulsed-Filed Gel Electrophoresis (PFGE) Results

**Figure 2:**
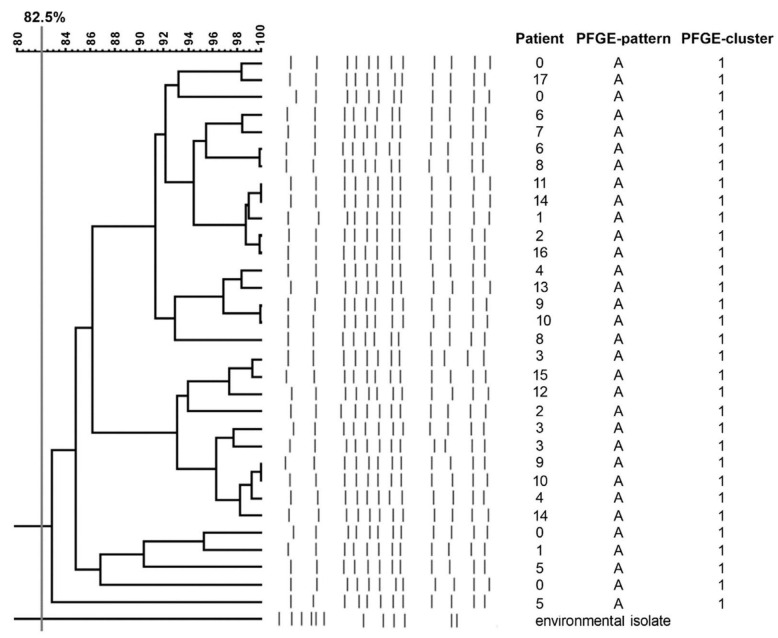
Pulsed-field gel electrophoresis of extended-spectrum β-lactamase-producing *Klebsiella pneumoniae* strains isolated from an outbreak in the Neonatal Clinic.

In total, 32 ESBL-KP isolates were investigated. All strains represented one clonal PFGE type (A) (Figure 2). *K. pneumoniae* isolated from an environmental source was not related to the outbreak strain and was designated as a unique PFGE pattern.

## 3. Discussion

An increasing frequency of *K. pneumoniae* emergence within the hospital environment has been reported worldwide. Moreover, the ability of the microorganism to adapt to complex hospital settings puts *K. pneumoniae* as the second most common agent of infection, especially in the intensive care unit (ICU) and neonatal intensive care unit (NICU). The rate of severe infections caused by *K. pneumoniae* in neonatal units is 18 to 68% [4]. In NICU, the rate of sepsis ranges between 28 and 50% of cases with a high prevalence of *Klebsiella* infections, as bloodstream infections are the primary type of infections [5]. In Italy, the percentage of infected newborns reached 14.1% [6]. In Germany, an epidemic outbreak involving 37 cases was described, of which 10 developed a bloodstream infection [7]. Our work confirmed three sepsis cases and nine other infections (UTI, pneumonia) during a two-month outbreak.

*K. pneumoniae* infections in neonatal wards are associated with low gestational age, low birth weight, invasive devices (mechanical ventilation or umbilical catheterization), or underlying conditions such as congenital disorders. We observed infections and cases of colonization in both low birth weight and term infants. Still, gestational age and Apgar scores were statistically lower in the group of infected neonates. Males appear to be significantly more likely to be infected and colonized with *K. pneumoniae*. This finding is supported by Haller’s study [7], with the percentage of infected males making up 60% of the neonates studied. In the outbreak we described, of the 18 neonates, 11 were male (61.1%), but it was not statistically significant.

*K. pneumoniae* is one of the first microorganisms to colonize neonatal intestines. Moreover, on par with *Escherichia coli*, it is a so-called “long-term” colonizer due to its diverse virulence factors that ensure its stable survival [8]. Most data on infections caused by *K. pneumoniae* preceded by colonization are associated with neonatal wards. Garrett et al. [9] demonstrated that early colonization of *K. pneumoniae* before other representatives of the microbiota appear is sufficient to initiate infection. However, Pope et al. [10] suggested a modulatory effect of *K. pneumoniae* on other components of the gut microbiota and that coexistence of this microbe with other bacteria limits the pathogenic potential of this pathogen. The presence of *K. pneumoniae* has been confirmed in anal swabs, umbilical cord, or oropharyngeal swabs of neonates hospitalized in NICU, which may support this observation. Bor and Ilhan [11] describe the most critical risk factors that significantly affect mortality among neonates infected by *K. pneumoniae*. These include congenital urinary tract abnormalities or respiratory tract immaturity. Although neonatal mortality resulting from sepsis caused by carbapenemase-negative *K. pneumoniae* is lower than that from carbapenemase-positive strains, it accounts for nearly a quarter of all deaths [12]. It is worth noting that the rate of neonatal infections caused by *K. pneumoniae* in developed and developing countries does not differ significantly [13]. However, the mortality rate is higher in the latter case. In our study, the most severe infections were observed in only three cases, and none of them was fatal.

Colonization of hospitalized neonates in a single ward is often associated with a single clonal strain, the transmission of which is facilitated mainly by healthcare personnel’s hand carriage during nursery, including casual contact [14]. Moreover, it is often predictive of infection and its prolonged duration and is closely associated with a higher risk of severe health consequences. Nordberg et al. [15] observed one and two-year-lasting colonization of *K. pneumoniae*, respectively, in one-fourth of infants who become colonized during previous hospitalizations. Moreover, long-term colonization with *K. pneumoniae* is also dependent on the type of delivery; neonates delivered by Cesarean section are initially exposed to hospital pathogens and are not colonized with the maternal vaginal or intestinal microbiota. The early introduction of antimicrobial therapy is also a contributing factor. It has also been proven that colonization screening of neonates in high-risk groups is a reliable procedure recommended for predicting infection and successfully controlling an outbreak [16]. Therefore, efforts should be immediately made to assess the colonization rate between currently hospitalized neonates. Early confirmation of colonization with antimicrobial susceptibility determination can support the infection risk assessment and the implementation of the appropriate empirical treatment strategy. In the present study, colonization with ESBL-KP was recognized in the case of six patients. In this group, no symptoms of infection were observed, and antimicrobial therapy was not implemented before the period of hospitalization. The administration of antibiotics to eradicate the colonization might not only increase the incidence of colonization but might also significantly affect the selection of multidrug-resistant strains [17]. Therefore, in the colonized neonates, only the inflammatory parameters were subsequently monitored in order to predict possible sequelae.

The emergence propensity of ESBL strains is associated with the widespread use of the third generation of cephalosporins, demonstrating the lack of activity against ESBL-producing bacteria. Treatment includes mainly carbapenems and non-β-lactam drugs; however, it is limited by the specificity of patients hospitalized in the neonatal intensive care unit (quinolones or tetracyclines exclusion). ESBL hydrolyzes most β-lactams; however, its activity is impaired by β-lactamase inhibitors. In our study, the first colonization ESBK-KP was confirmed on the day of admission in the index case (patient „0”). Nevertheless, all our outbreak strains were resistant to piperacillin with tazobactam, which may be the consequence of TEM β-lactamase overproduction [18].

ESBL production is strongly related to virulence factor expression, which promotes intestinal colonization. Moreover, colonization with ESBL-KP lasts longer. Virulence of *K. pneumoniae* may have a destructive influence on the neonate’s organism, leading to the hematogenous spread of primary infection. Moreover, Fasciana et al. [19] described a strong association between virulence and multidrug resistance in *K. pneumoniae*. Such a relationship is related to the spread of resistant strains carrying many genes encoding virulence factors strongly conserved with certain clonal types. Furthermore, *K. pneumoniae* virulence factors—such as polysaccharide capsule or siderophore production—enable successful colonization of patients and facilitate the survival of *K. pneumoniae* on inanimate objects, making it much more resistant to standard infection prevention practices; therefore, successful outbreak control depends on effective internal communication strategy [20]. In this study, all strains carried genes related to adhesion, iron uptake, and lipopolysaccharide (LPS) production.

PFGE remains the preferred typing method for many strictly epidemiological applications [21]. The choice of this genotyping method in our study can also be supported by the fact that only a limited number of strains exist. PFGE method allows determining the dissemination of clonally related strains in patients and the environment and has a tremendous discriminatory power to determine the *K. pneumoniae* outbreak. Broad consistencies between *K. pneumoniae* colonizing patients and the ones isolated from outbreaks are observed in multiple studies, where outbreaking strains are mainly classified into one epidemic clone, proving its shared origin spread on the hands of healthcare personnel or transmission via medical devices [22]. The emergence of *K. pneumoniae*, one clonal type found within the patients without causing the disease, demonstrated this microorganism’s excellent colonization capability. It was confirmed by studies conducted in Germany, Italy, and Poland [6,7,23]. Similarly, PFGE results indicated the clonal nature of all ESBL-KP strains isolated from our neonates, confirming their horizontal transfer during the outbreak.

It is well known that the main reservoir of *K. pneumoniae* is the human gastrointestinal tract. Rapid acquisition of resistance to multiple antimicrobial agents and emergence allows *K. pneumoniae* to persist in the hospital environment and on invasive diagnostic tools. Furthermore, the bacterium readily colonizes the hands of individuals exposed to hospital settings (e.g., staff, mothers). Unidentified carriers colonized with *K. pneumoniae* are an essential vector of horizontal pathogen transmission, facilitating the direct transmission of this microorganism to patients. Nearly 40% of neonatal infections caused by *K. pneumoniae* are estimated to be endogenous, whereas exogenous infections are associated with the hand transmission of *K. pneumoniae* by colonizing healthcare personnel [24]. Development, implementation, and supervision of infection prevention procedures, including decontamination of skin and mucous membranes, are established by the Infection Control Team each time, with consideration for the size of the outbreak and the situation following the implementation of intensified decontamination procedures [25]. In the present study, the most probable reservoir of the ESBL-KP outbreak was an 11-day-old male neonate (patient „0”), transferred to NC from the obstetrics/gynecology ward of a provincial hospital with positive ESBL-KP oral/rectum swabs taken at admission. The patient was isolated, and the following ESBL-KP-positive cases also were isolated/cohorted. Implementing enhanced comprehensive infection control measures involved strict handwashing, frequently changing gloves, reducing, to a minimum, the number of staff visiting patients, intensifying environmental control, and decontamination. All environmental controls showed the lack of an ESBL-KP outbreak. The lack of ESBL-KP in the environment suggests that the outbreak was transmitted by colonized hospital staff. Nevertheless, procedures to determine colonization in healthcare personnel were not implemented, and the colonization rate among the hospital staff was not determined. However, the epidemiological measures taken have been successful to some extent. No new cases of investigated ESBL-KP infection or colonization were confirmed in the 6 months following the last confirmed case of colonization.

## 4. Materials and Methods

### 4.1. Hospital Settings

The outbreak occurred in a tertiary teaching hospital in Szczecin, Poland comprising a Neonatal Clinic with 30 beds in 11 rooms, including intensive care, pathology, and isolates units, ranging from one to four beds per room and four beds per nurse. Neonates are admitted to the NC, principally from the labor ward. Occasionally, neonates are transferred from other hospitals. According to the Hospital Infection Control Committee (HICC) guidelines, the surveillance of ESBL-KP rectal colonization is routinely screened once a week in high-risk neonates hospitalized in NC, all with symptoms of infection and in all transferred from another hospital. Neonates identified as ESBL-KP carriers or infected (or both) are isolated/cohorted and managed by individual nursing staff. Infection control measures, including hand hygiene, cleaning, and disinfection protocols, also clinical and microbiological data are analyzed quarterly. ESBL-KP colonization or infection among neonates was observed occasionally. During the 6 months preceding the outbreak, three cases of colonization and one case of infection (UTI) were recognized, and 58 neonates were hospitalized. Antibiotic protocols and microbiological methods were not changed; routine environmental sampling was performed.

The institutional ethics committee authorized this retrospective report, which was planned after the outbreak and did not identify any patient.

### 4.2. Microbiological Diagnostic

An ESBL-KP outbreak occurred in NC between 10 October and 7 December 2019 (data of the first and the last positive ESBL-KP culture). In neonates with symptoms or suspicion of infection, blood, urine, and other samples were taken, and for all neonates, microbiological screening was performed. Positive ESBL-KP cultures were obtained from 18 neonates and one isolate from 42 environmental samples. The healthcare personnel had no hand and other specimens taken. Specimens were seeded according to HICC’s guidelines onto the following media: blood agar, chocolate agar, MacConkey agar, or ChromID ESBL agar (bioMerieux, Marcy-l’Étoile, France). *K. pneumoniae* was identified using VITEK 2 Compact (bioMerieux, Marcy-l’Étoile, France). Antimicrobial susceptibility testing (AST) for ampicillin, amoxicillin–clavulanate, piperacillin–tazobactam, meropenem, cefotaxime, ceftazidime, gentamicin, amikacin, and trimethoprim–sulfamethoxazole was performed using Kirby-Bauer agar disk diffusion method according to EUCAST guidelines [26]. The presence of ESBL was confirmed with the double disk method.

### 4.3. Molecular Typing

All ESBL-KP isolates were analyzed using macrorestriction with *XbaI* (Thermo Scientific, Waltham, MA, USA), and subsequent pulsed-field gel electrophoresis (PFGE) was performed according to the protocol described by the manufacturer. The gel was visualized under UV light and documented using Quantity One (Bio-Rad, Marnes-la-Coquette, France). Digital images were analyzed with FPQuest Software 4.5 (Bio-Rad, Marnes-la-Coquette, France). The dendrogram was generated using the Dice correlation coefficient and the unweighted pair group method with arithmetic mean and 2% band tolerance.

The classification of individual restriction patterns for particular genetic profiles was carried out using the Unweighted Pair Group Method with Arithmetic Mean (UPGMA) method (SAB value = 82.5%) and the Dice coefficient (2.0%).

### 4.4. PCR

PCR was performed using primer sequences specific for genes encoding ESBL enzymes (*bla*_TEM_, *bla*_SHV_, *bla*_OXA_, *bla*_CTX-M_) and selected virulence factors: fimbriae and mediating bacterial adhesion (*fimH*), genes involved in the production of LPS, which protects bacteria from the action of complement (*wabg*, *uge*) and genes determining iron acquisition (*kfu* and *iroN*).

The amplification reaction consisted of 30 cycles and was performed in an Applied Biosystems Veriti 96 Well Thermal Cycler (Applied Biosystems, Norwalk, CT, USA). Primers and parameters used in the study were previously described [27,28].

Electrophoresis was performed using a 1.5% agarose gel (DNA Gdansk, Gdansk, Poland) mixed with 0.5 μg/mL ethidium bromide (Sigma-Aldrich, Darmstadt, Germany). The result was read under UV light using the GelDoc-It2 Imager system (Upland, CA, USA). Positive controls were standard strains suitable for specific genes.

### 4.5. Statistics

Data were organized and analyzed using GraphPad Prism 5.02 (GraphPad Software Inc., San Diego, CA, USA) software. Then, they were analyzed with the nonparametric tests (Chi-square continuity test with Yates correction or Mann–Whitney U test). Statistical significance was set as a *p* < 0.05.

## 5. Conclusions

Lower Apgar score and birth age were significant risk factors for ESBL-KP infection. The retrospectively described outbreak involved 18 neonates, of which 12 were infected and six were merely/only colonized with an ESBL-KP strain belonging to a single genetic clone. Immediate tightening of hospital hygiene rules, screening of all hospitalized neonates, and isolation/cohorting of ESBL-KP-positive patients proved effective in controlling and finishing the two-month outbreak. The lack of ESBL-KP in the environment supposes that the outbreak was transmitted by colonized hospital staff. Nevertheless, the colonization rate of hospital staff was not determined. The colonization screening of hospital staff might probably provide further details about the transmission pathway and successful outbreak control.

## Figures and Tables

**Table 1:** Characteristic of neonates with laboratory-confirmed extended-spectrum β-lactamase-producing *Klebsiella pneumoniae* during the outbreak (between 10 October and 7 December 2019).

Patient No.	Date of Birth	Date of 1. ESBL-KP Isolation	Sex	Pregnancy	Childbirth	HBD	Apgar Score	Birth Weight (g)	Assisted Ventilation	Antibiotic Therapy	Colonization Specimen	Infection Specimen	Clinical Diagnosis
„0”	9 Sep	10 Oct	M	C	CS	38	10,10,10	2930	no	yes	OC,R	-	PNA
1.	20 Oct	26 Oct	M	N	CS	38	6,7,7	4200	yes	yes	R	CON	CONS, PNA
2.	21 Oct	26 Oct	M	N	NAT	37	9,9,9	3130	yes	yes	R	B,U,CSF	PNA, UROSEP, MEN
3.	27 Oct	2 Nov	M	N	CS	35	6,8,9	1880	no	yes	R	B	SEP
4.	29 Oct	3 Nov	F	C	CS	33	8,8,8	2230	no	yes	OC,R	U	UTI
5.	28 Oct	4 Nov	F	N	CS	37	8,8,8	2000	no	no	OC,R	-	COL
6.	21 Oct	7 Nov	F	C	NAT	30	7,8,9	1460	yes	yes	R	B	SEP,PNA
7.	3 Nov	8 Nov	M	C	CS	35	10,10,10	1700	no	yes	OC,R	-	PNA
8.	4 Nov	8 Nov	M	C	CS	39	10,10,10	3400	yes	yes	R	-	PNA
9.	4 Nov	8 Nov	F	C	CS	35	9,9,10	2450	no	no	OC,R	-	COL
10.	13 Oct	13 Nov	F	C	CS	28	2,3,5	1080	yes	yes	R	-	PNA
11.	14 Oct	13 Nov	M	N	CS	29	6,6,7	1250	yes	yes	R	-	PNA
12.	12 Nov	21 Nov	F	C	NAT	40	10,10,10	3470	no	no	R	-	COL
13.	19 Nov	21 Nov	F	C	CS	39	10,10,10	2300	no	no	R	-	COL
14.	8 Nov	21 Nov	M	C	CS	35	9,9,9	3080	yes	yes	OC,R	-	PNA
15.	21 Nov	23 Nov	M	C	CS	39	8,9,10	2800	no	no	R	-	COL
16.	21 Oct	23 Nov	M	C	CS	39	8,9,10	4110	no	no	R	-	COL
17.	4 Nov	7 Dec	M	N	NAT	38	8,8,8	3180	no	yes	R	U	UTI

M—male, F—female, N—normal, C—complicated, CS—Cesarean section, NAT—natural childbirth, OC—oral cavity; R—rectum; CSF—cerebrospinal fluid, CON—conjunctiva; CONS—conjunctivitis; B—blood; U—urine; UTI—urinary tract infection, PNA—pneumonia; SEP—sepsis; UROSEP—urosepsis; MEN—meningitis; COL—colonization; HBD—hebdomas graviditatis.

**Table 2:** Risk factors of extended-spectrum β-lactamase-producing *Klebsiella pneumoniae* colonized and infected neonates.

Variable	Descriptive Statistic	Colonized (*n* = 6)	Infected (*n* = 12)	*p*-Value (Univariate)
Gender (male)	*n* (%)	2 (33.3)	9 (75.0)	0.232
Birth weight (g)	median (IQR)	2625 (2338–3303)	2580 (1640–3143)	0.437
Gestational age (week)	median (IQR)	39 (37.5–39)	35 (32–38)	0.022
Apgar score	median (IQR)	9.5 (8–10)	8 (7–9)	0.016
Gestational course (complicated)	*n* (%)	5 (83.3)	7 (58.3)	0.596
Cesarean section (yes)	*n* (%)	5 (83.3)	9 (75.0)	0.841
Assisted ventilation	*n* (%)	0 (0.0)	7 (58.3)	0.060

IQR—interquartile range.

## Data Availability

Not applicable.
